# Comparison of Estimated Rates of Coronavirus Disease 2019 (COVID-19) in Border Counties in Iowa Without a Stay-at-Home Order and Border Counties in Illinois With a Stay-at-Home Order

**DOI:** 10.1001/jamanetworkopen.2020.11102

**Published:** 2020-05-15

**Authors:** Wei Lyu, George L. Wehby

**Affiliations:** 1 Department of Health Management and Policy, The University of Iowa, Iowa City; 2 Department of Economics, The University of Iowa, Iowa City; 3 Department of Preventive and Community Dentistry, The University of Iowa, Iowa City; 4 Public Policy Center, The University of Iowa, Iowa City; 5 National Bureau of Economic Research, Cambridge, Massachusetts

## Abstract

**Importance:**

Iowa is 1 of 5 states in the US that have not issued a stay-at-home order during the coronavirus disease 2019 (COVID-19) pandemic. There is no empirical evidence on whether issuing a stay-at-home order in Iowa could have been associated with a reduced rate of COVID-19 infections in the state.

**Objective:**

To compare COVID-19 cases in border counties in Iowa, which did not issue a stay-at-home order, with cases in border counties in Illinois, which did issue a stay-at-home order.

**Design, Setting, and Participants:**

This cross-sectional study with a difference-in-differences design compared daily changes in COVID-19 cases per 10 000 residents in 8 Iowa counties bordering Illinois with those in the 7 Illinois counties bordering Iowa before and after Illinois issued a stay-at-home order on March 21, 2020. Additional sensitivity analyses were conducted to account for differences in timing of closing schools and nonessential businesses between the 2 states and differential trends in COVID-19 cases by county population density and poverty rates.

**Exposures:**

Issuing a stay-at-home order.

**Main Outcomes and Measures:**

Comparison of cumulative cases of COVID-19 per 10 000 residents in border counties in Iowa and Illinois.

**Results:**

The total populations were 462 445 in the Iowa border counties and 272 385 in the Illinois border counties. Population density was higher in the Iowa counties (114.2 people per square mile) than in the Illinois counties (78.2 people per square mile). Trends of cumulative COVID-19 cases per 10 000 residents for the Iowa and Illinois border counties were comparable before the Illinois stay-at-home order, which went into effect at 5:00 pm on March 21 (March 15 to March 21: 0.024 per 10 000 residents vs 0.026 per 10 000 residents). After that, cases increased more quickly in Iowa and more slowly in Illinois. Within 10, 20, and 30 days after the enactment of the stay-at-home order in Illinois, the difference in cases was −0.51 per 10 000 residents (SE, 0.09; 95% CI, −0.69 to −0.32; *P* < .001), −1.15 per 10 000 residents (SE, 0.49; 95% CI, −2.12 to −0.18; *P* = .02), and −4.71 per 10 000 residents (SE, 1.99; 95% CI, −8.64 to −0.78; *P* = .02), respectively. The estimates indicate excess cases in the border Iowa counties by as many as 217 cases after 1 month without a stay-at-home order. This estimate of excess cases represents 30.4% of the 716 total cases in those Iowa counties by that date. Sensitivity analyses addressing differences in timing of closing schools and nonessential businesses and differences in county population density and poverty rates between the 2 states supported these findings.

**Conclusions and Relevance:**

This cross-sectional study with a difference-in-differences design found an increase in estimated rates of COVID-19 cases per 10 000 residents in the border counties in Iowa compared with the border counties in Illinois following a stay-at-home order that was implemented in Illinois but not in Iowa.

## Key Points

**Question:**

Was the stay-at-home order in Illinois associated with different rates of coronavirus disease 2019 (COVID-19) compared with Iowa, which did not issue a stay-at-home order?

**Findings:**

This cross-sectional study of border counties in Iowa and Illinois used difference-in-differences design and found an increase in estimated rates of COVID-19 cases per 10 000 residents in the border counties in Iowa compared with the border counties in Illinois after a stay-at-home order was implemented in Illinois but not in Iowa.

**Meaning:**

The results of this study suggest that issuing a stay-at-home order in Iowa may have helped limit the spread of COVID-19 cases in that state.

## Teaser

This cross-sectional study compares coronavirus disease 2019 (COVID-19) cases in border counties in Iowa, which did not issue a stay-at-home order, with cases in border counties in Illinois, which did issue a stay-at-home order.

## Introduction

Iowa is 1 of 5 states that have not issued stay-at-home orders for the coronavirus disease 2019 (COVID-19) pandemic. The state has issued a series of orders, including banning large gatherings and closing bars and restaurant dining on March 17, 2020; closing some nonessential businesses (eg, dental offices, clothing stores, barbershops, massage therapy, medical spas) on March 26, 2020; closing all primary and secondary schools (recommended on March 15, 2020) on April 2, 2020; and closing additional businesses (eg, malls, nongrocery stores, museums, libraries, social clubs) on April 6, 2020.^1^

Multiple constituents have petitioned Iowa’s governor to issue a stay-at-home order (including the Iowa Board of Medicine).^2^ However, the governor has indicated that the existing restrictions are essentially equivalent to stay-at-home orders in other states.^3^ There is no empirical evidence on whether issuing a stay-at-home order in Iowa could have been associated with a reduced rate of COVID-19 infections in the state. Illinois, which borders Iowa, issued a stay-at-home order effective on the evening of March 21, 2020.^4^ The order also closed all nonessential businesses in that state. Using a cross-sectional study with difference-in-differences design, we compared the cases of COVID-19 in border counties in Iowa and Illinois.

## Methods

This cross-sectional study used a difference-in-differences design to compare daily changes in COVID-19 cases per 10 000 residents in 8 Iowa counties bordering Illinois (ie, Clinton, Des Moines, Dubuque, Jackson, Lee, Louisa, Muscatine, and Scott) with those in 7 Illinois counties bordering Iowa (ie, Carroll, Hancock, Henderson, Jo Daviess, Mercer, Rock Island, and Whiteside) before and after Illinois issued a stay-at-home order on March 21, 2020. County-level COVID-19 data come from a repository of state and local health agency reports.^5^ The difference-in-differences design assumes similarity of COVID-19 trends across the border if Iowa and Illinois had issued similar orders. We evaluated this assumption by comparing COVID-19 trends before enacting the order. The study followed the Strengthening the Reporting of Observational Studies in Epidemiology (STROBE) reporting guideline for cross-sectional studies. Per the Common Rule, institutional review board review was not required for this study, which used deidentified, publicly available data.

### Statistical Analyses

We estimated the following difference-in-differences regression using daily county-level COVID-19 data: *Cases_ct_* = α + *β_1_StayHome_c_* × *Post_t_* + *θ_c_* + *ω_t_* + *ϵ_ct_*, in which *β_1_* is the difference-in-differences estimate, representing the pre-post stay-at-home order differential change in COVID-19 cases per 10 000 residents (*Cases)* between Iowa and Illinois, *c* indicates a specific county, and *t *indicates a specific day. The pre-period is from March 15 (when the first case was reported in those counties) to March 21. We performed a *t* test to test whether the *β_1_* equaled 0 at a significance level of *P* < .05. We estimated this model separately for 6 post-periods (each 5 days) from March 22 (the first full day with a stay-at-home order in Illinois) until April 20. County-specific fixed effects (*θ_c_*) captured county time-invariant differences, such as population density and demographic and socioeconomic characteristics, which should not change during the short study period. Day fixed effects (*ω_t_*) captured daily changes in COVID-19 spread shared between Iowa and Illinois. The regression was estimated via least squares weighted by 2019 county population. We tested for homoscedasticity using a Breusch-Pagan test; the test rejected homoscedasticity at *P* < .001. Therefore, we estimated heteroscedasticity-robust standard errors.

We also performed 2 sensitivity analyses. The first sensitivity analysis accounted for differences in the timing of closing schools and nonessential businesses between the 2 states. We reestimated the regressions adding 2 state-level time-varying indicators as covariates, as follows: (1) whether the state had issued an order for closing primary and secondary schools by that day (issued on April 2 in Iowa and March 17 in Illinois) and (2) whether the state had issued an order to close nonessential businesses (the first order in Iowa was on March 26; in Illinois, closure of nonessential businesses was part of the stay-at-home order, so it was assigned the same date). The second sensitivity analysis examined whether there were differential trends in COVID-19 cases by county population density and poverty rates, reported by census data,^6^ that may have confounded the difference-in-differences estimates. For this analysis, we added an interaction between the county population density and the post-period indicator (post) and another interaction between the county poverty rate and post to each regression.

To address testing rate differences between the counties in Iowa vs Illinois, we examined daily state level testing data from the COVID Tracking Project to determine total tests per 10 000 residents in Iowa and Illinois.^7^ Data analyses were conducted with Stata/SE version 16.0 (StataCorp). Statistical significance was set at *P* < .05, and all tests were 2-tailed.

## Results

The total population (based on 2019 census estimates) in the 8 Iowa border counties was 462 445; the total population in the 7 Illinois border counties was 272 385.^8^ Population density was higher in the Iowa counties (114.2 people per square mile) vs the Illinois counties (78.2 people per square mile).^8,9^ The population poverty rate was slightly higher in the Iowa counties (12.1% vs 10.8%).^6^

**Figure 1.**
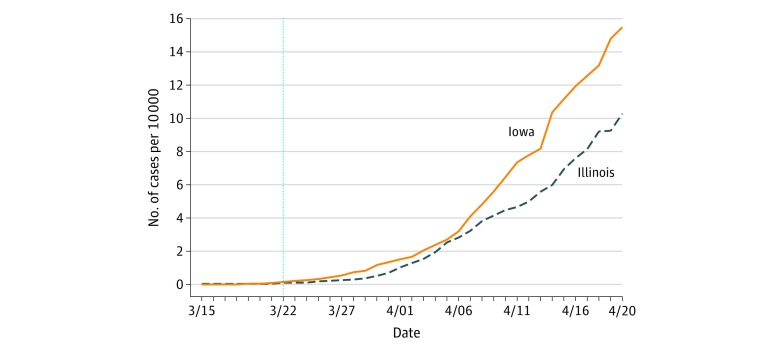
Cumulative Coronavirus Disease 2019 (COVID-19) Cases per 10 000 Residents in Iowa and Illinois Border Counties The vertical line represents the date on which the stay-at-home order took effect in Illinois.

Figure 1 shows daily cumulative COVID-19 cases per 10 000 residents for the Iowa and Illinois border counties. Trends were comparable before the Illinois stay-at-home order became effective, supporting the difference-in-differences design. From March 15 to March 21 (on the evening of which the stay-at-home order became effective in Illinois), the average daily cases per 10 000 residents was 0.024 in the Iowa counties and 0.026 in the Illinois counties. After that, cases increased more quickly in Iowa. The difference-in-differences regression estimates also indicated a slower increase in COVID-19 cases in Illinois (Table 1). Within 10, 20, and 30 days after the enactment of the stay-at home order in Illinois, the difference in cases was −0.51 per 10 000 residents (SE, 0.09; 95% CI, −0.69 to −0.32; *P* < .001), −1.15 per 10 000 residents (SE, 0.49; 95% CI, −2.12 to −0.18; *P* = .02), and −4.71 per 10 000 residents (SE, 1.99; 95% CI, −8.64 to −0.78; *P* = .02), respectively. The estimates indicate excess cases in the border Iowa counties by as many as 217 after 1 month without a stay-at-home order. This estimate of excess cases represents 30.4% of the 716 total cases in those Iowa counties by that date.

**Table 1.** Difference-in-Differences Estimates of COVID-19 Cases Comparing Border Counties in Iowa With Those in Illinois Before and After the Stay-at-Home Order Was Issued in Illinois^a^

Period	Difference in COVID-19 cases per 10 000 residents^b^	Heteroskedasticity robust SE (95% CI)^c^	*P* value	Excess cases in Iowa border counties	Excess cases as proportion of total cases, %
3/22-3/26	−0.14	0.04 (−0.23 to −0.06)	.001	6	32.4
3/27-3/31	−0.51	0.09 (−0.69 to −0.32)	<.001	24	38.0
4/01-4/05	−0.41	0.17 (−0.74 to −0.07)	.02	19	15.2
4/06-4/10	−1.15	0.49 (−2.12 to −0.18)	.02	53	17.8
4/11-4/15	−3.35	1.19 (−5.70 to −0.99)	.006	154	30.0
4/16-4/20	−4.71	1.99 (−8.64 to −0.78)	.02	217	30.4

Abbreviation: COVID-19, coronavirus disease 2019.

^a^ The regression model was estimated separately for each of 5-day period. The regression was estimated using least squares weighted by the 2019 county population. The regression adjusted for county and day fixed effects. The number of county-day observations was 180 for each regression.

^b^ This indicates the estimated difference-in-differences association of a stay-at-home order with COVID-19 cases in a given period relative to March 15 to March 21 (ie, the period before the stay-at-home order in Illinois was enacted).

^c^ Heteroskedasticity robust SEs were estimated because homoscedasticity is rejected for all post-period regressions.

In the first sensitivity analysis accounting for differences in the timing of closing schools and nonessential businesses between the 2 states, the difference-in-differences estimates for the stay-at-home order were similar, although not statistically significant at 20 and 30 days (eg, difference in COVID-19 cases per 10 000 residents in Illinois at 10 days, −0.51; SE, 0.10; 95% CI, −0.71 to −0.31; *P* < .001; 20 days, −1.18; SE, 0.61; 95% CI, −2.39 to 0.04; *P* = .06; 30 days, −4.73; SE, 2.57; 95% CI, −9.81 to 0.35; *P* = .07) (Table 2). In the second sensitivity analysis examining whether there were differential trends in COVID-19 cases by county population density and poverty rates that may have confounded the difference-in-differences estimates, the difference-in-differences estimates were robust to adding these covariates (eg, difference in COVID-19 cases per 10 000 residents in Illinois at 10 days, −0.50; SE, 0.10; 95% CI, −0.70 to −0.30; *P* < .001; 20 days, −1.13; SE, 0.53; 95% CI, −2.18 to −0.77; *P* = .04; 30 days, −4.80; SE, 2.517; 95% CI, −9.09 to −0.52; *P* = .03) (Table 2).

**Table 2.** Sensitivity Analysis of Difference-in-Differences Estimates of COVID-19 Cases Comparing Border Counties in Iowa With Those in Illinois Before and After the Stay-at-Home Order Was Issued in Illinois^a^

Periods	Difference in COVID-19 cases per 10 000^b^	Heteroskedasticity robust SE (95% CI)^c^	*P* value
**Sensitivity check 1: controlling for closing schools and nonessential businesses**			
3/22-3/26	−0.14	0.06 (−0.26 to −0.03)	.01
3/27-3/31	−0.51	0.10 (−0.71 to −0.31)	<.001
4/01-4/05	−0.44	0.18 (−0.79 to −0.09)	.01
4/06-4/10	−1.18	0.61 (−2.39 to 0.04)	.06
4/11-4/15	−3.37	1.50 (−6.33 to −0.41)	.03
4/16-4/20	−4.73	2.57 (−9.81 to 0.35)	.07
**Sensitivity check 2: controlling for differential trends in population density and poverty rate**			
3/22-3/26	−0.14	0.05 (−0.23 to −0.05)	.003
3/27-3/31	−0.50	0.10 (−0.70 to −0.30)	<.001
4/01-4/05	−0.40	0.18 (−0.76 to −0.04)	.03
4/06-4/10	−1.13	0.53 (−2.18 to −0.08)	.04
4/11-4/15	−3.37	1.32 (−5.97 to −0.77)	.01
4/16-4/20	−4.80	2.17 (−9.09 to −0.52)	.03

Abbreviation: COVID-19, coronavirus disease 2019.

^a^ The regression model was estimated separately for each of 5-day period. The regression was estimated using least squares weighted by the 2019 county population. The regression adjusted for county and day fixed effects. The number of county-day observations was 180 for each regression.

^b^ This indicates the estimated difference-in-differences association of a stay-at-home order with COVID-19 cases in a given period relative to March 15 to March 21 (ie, the period before the stay-at-home order in Illinois was enacted).

^c^ Heteroskedasticity robust SEs were estimated because homoscedasticity is rejected for all post-period regressions.

**Figure 2.**
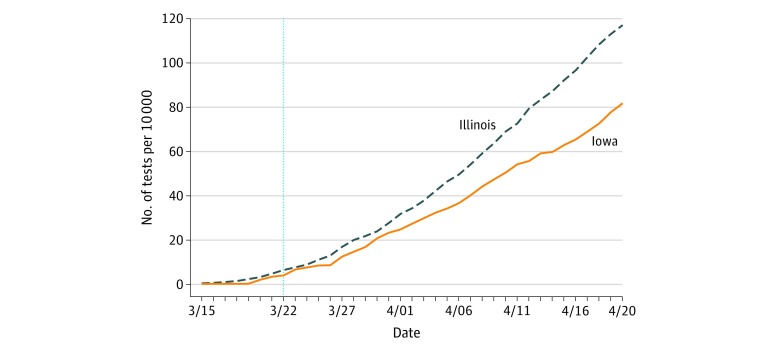
Statewide Cumulative Coronavirus Disease 2019 Tests per 10 000 Residents in Iowa and Illinois The vertical line represents the date on which the stay-at-home order took effect in Illinois.

Illinois had a greater increase in tests per 10 000 residents following the stay-at-home order. Both states started with a similar testing rate, but the testing rate in Illinois increased after the stay-at-home order at a faster rate than in Iowa (10 days, 28 vs 23 tests per 10 000 residents; 20 days, 69 vs 51 tests per 10 000 residents; 30 days, 117 vs 82 tests per 10 000 residents) (Figure 2).

## Discussion

This cross-sectional study with difference-in-differences design found an increase in estimated rates of COVID-19 cases per 10 000 residents in the border counties in Iowa compared with the border counties in Illinois following a stay-at-home order that was implemented in Illinois but not in Iowa. The estimates indicated as many as 217 excess cases in Iowa after 1 month without a stay-at-home order. Our findings are also consistent with recent evidence from California,^10^ where a statewide stay-at-home order was associated with reduced COVID-19 cases by 125.5 to 219.7 per 100 000 residents by April 20. Our findings suggest that issuing a stay-at-home order in Iowa while daily cases continued to increase may have helped limit the number of cases.

In the last 2 days of the study period (ie, April 19-20), Iowa began announcing an increase in cases from surveillance testing following outbreaks in meat-processing facilities, including 1 facility in Louisa County.^11^ That outbreak appears to have been first reported on April 6.^12^ As described earlier, there was a trend of more cases in Iowa before April 6 and the trend increased during most of the study period. Therefore, the surveillance testing reported toward the end of the study period does not appear to explain the whole trend. Meat-processing plants would not be closed during a stay-at-home order because they are considered essential businesses. Whether stay-at-home orders could modify the likelihood of such outbreaks (through initial cases) and their subsequent effects on community spread is unknown and is an important question for future research, given the outbreaks reported in multiple meat-processing facilities in Iowa and other states.

### Limitations

This study has limitations. Findings should be interpreted cautiously considering that possible differences in COVID-19 testing across the border may confound the difference-in-differences estimates. For example, Illinois had a greater increase in tests per 10 000 residents after the stay-at-home order (Figure 2). If state-level testing rates are representative of these counties, this would suggest that Iowa’s testing rate was not higher during this period, and therefore the difference in testing would not explain the observed difference in rates per capita after the stay-at-home order. However, county-level testing trends may be different from state trends and may still confound the difference-in-differences estimates. As noted earlier, a meat-packing facility in Iowa had an outbreak, which increased surveillance. Whether stay-at-home orders affect the likelihood of such outbreaks and their spillovers into the community is an open question for future research.

## Conclusions

This cross-sectional study of counties along the border of Iowa and Illinois provides early evidence suggesting that issuing a stay-at-home order in Iowa while daily cases continued to increase may have helped limit the spread of COVID-19 cases in Iowa. Further research is needed to examine the effects of stay-at-home orders in other areas and potential heterogeneity by population and contextual factors.
